# Exploring genetic testing requests, genetic alterations and clinical associations in a cohort of children with autism spectrum disorder

**DOI:** 10.1007/s00787-024-02413-x

**Published:** 2024-04-08

**Authors:** Nathalia Garrido-Torres, Renata Marqués Rodríguez, María Alemany-Navarro, Javier Sánchez-García, Susana García-Cerro, María Irene Ayuso, Antonio González-Meneses, Amalia Martinez-Mir, Miguel Ruiz-Veguilla, Benedicto Crespo-Facorro

**Affiliations:** 1 Instituto de Biomedicina de Sevilla, Seville, Spain; 2 University of Seville, Seville, Spain; 3 CIBERSAM, ISCIII (Spanish Network for Research in Mental Health), Seville, Spain; 4 Hospital Universitario Virgen del Rocío, Seville, Spain; 5 Department of pediatrics, Hospital La Merced de Osuna, Seville, Spain; 6 Department of Maternofetal Medicine, Genetics and Reproduction, Seville, Spain; 7 Spanish National Research Council (CSIC), Seville, Spain; 8 Center for Biomedical Network Research on Rare Diseases (CIBERER), Seville, Spain

**Keywords:** Autism spectrum disorder, Neurodevelopmental disorders, Genetics, Child psychiatry, Diagnosis

## Abstract

Several studies show great heterogeneity in the type of genetic test requested and in the clinicopathological characteristics of patients with ASD. The following study aims, firstly, to explore the factors that might influence professionals’ decisions about the appropriateness of requesting genetic testing for their patients with ASD and, secondly, to determine the prevalence of genetic alterations in a representative sample of children with a diagnosis of ASD. Methods: We studied the clinical factors associated with the request for genetic testing in a sample of 440 children with ASD and the clinical factors of present genetic alterations. Even though the main guidelines recommend genetic testing all children with an ASD diagnosis, only 56% of children with an ASD diagnosis were genetically tested. The prevalence of genetic alterations was 17.5%. These alterations were more often associated with intellectual disability and dysmorphic features. There are no objective data to explicitly justify the request for genetic testing, nor are there objective data to justify requesting one genetic study versus multiple studies. Remarkably, only 28% of males were genetically tested with the recommended tests (fragile X and CMA). Children with dysmorphic features and organic comorbidities were more likely to be genetic tested than those without. Previous diagnosis of ASD (family history of ASD) and attendance at specialist services were also associated with Genetically tested Autism Spectrum Disorder GTASD. Our findings emphasize the importance of establishing algorithms to facilitate targeted genetic consultation for individuals with ASD who are likely to benefit, considering clinical phenotypes, efficiency, ethics, and benefits.

**Supplementary Information:**

The online version contains supplementary material available at 10.1007/s00787-024-02413-x.

## Introduction

Autism Spectrum Disorder (ASD) is a neurodevelopmental disorder (NDD) that is characterised by difficulties in communication and social interaction, and the presence of repetitive behaviours and interests [1–3]. ASDs share many clinical features with other NDDs, and most also have deficits or delays in other areas of neurodevelopment and comorbidities with other psychiatric disorders [4–6]e.g. more than 50% may present concurrent physical or mental conditions [2].In recent years, the prevalence of ASD patients has shown an increase [4, 7, 8], and it also varies across different series and countries [9, 10]. A recent study in the US revealed a prevalence of 2.3% [4], while in Spain it is estimated to range from 0.7–1.5% [8, 9, 11]. This data indicates an increase in newly diagnosed autism cases in Spain between 2009 and 2017. The causes of this increase are to some extent justified by a better knowledge of its pathophysiology and increasingly better established diagnostic criteria [7], currently using by consensus the criteria established by the DSM-5 [3] and ICD-11 [12]. Families receiving a diagnosis of autism are typically offered a range of recommendations, which may include educational, behavioural and medical community resources, including genetic testing.

There have also been major advances in the aetiological and, in particular, genetic diagnosis of ASD in recent years. In families with a child with autism, the risk of recurrence increases 8.4-fold for siblings and 2-fold for cousins [13]. This fact reflects the key role of genetics in the aetiology of ASD, with heritability estimated at 65–90% [14, 15]. Autism is linked to various genetic disorders, yet only a small portion can be attributed to a single gene cause. For instance, fragile X syndrome and tuberous sclerosis, collectively, are estimated to contribute to less than 10% of autism cases, although 50–70% of individuals with fragile X syndrome display symptoms of ASD. More commonly, heterozygous *de novo* single nucleotide variants (SNVs) and/or abnormalities in submicroscopic segments of DNA called copy number variations (CNVs) are found, with over 100 genes associated with autism having been identified and described in multiple studies over the last decade [16, 17]. Some studies have found that using chromosomal microarray analysis (CMA), approximately 10-20% of autistic individuals receive a molecular diagnosis [18–21]. CMA for CNVs has been recommended as the first genetic test for children with NDDs [22–24].

The American College of Medical Genetics and Genomics (ACMG) guidelines also recommend CMA for all children with ASD, fragile X testing for males and additional genetic sequencing, including *PTEN* gene in children with macrocephaly and *MECP2* gene in girls with psychomotor regression [25]. However, there is clear variation in the utilization of genetic testing within the etiological algorithm of ASD diagnosis. Another recent studies have demonstrated an increased diagnostic yield with exome sequencing, with percentages that vary from 39% [21] to 75% [26] compared to 10–20% with CMA. Consequently, exome sequencing has been proposed as the initial diagnostic test. Srivastava (2019) [21] and Satterstrom et al. (2020) [27] conducted the first large-scale studies utilizing exome sequencing in ASD patients, identifying 102 risk genes, many of which are expressed in the brain, underscoring the extensive genetic heterogeneity present in ASD. Although the yield is greater in the exome, there are ethical-legal implications and potential risks. Therefore, your request must have a clinical justification. The American Academy of Pediatrics recommends considering exome sequencing if other testing modalities yield negative results [28].

Several studies show great heterogeneity in the type of testing requested and the clinicopathological characteristics of the patients [29–31]. It is known that genetic alteration is not necessarily associated with dysmorphic features and organic comorbidities [32]. The autistic children who got genetic referrals were more likely to have intellectual disability and language disorder [33].

The utilization of genetic testing for ASD varies globally, revealing discrepancies in accessibility and implementation. Studies conducted in the USA by Harris et al. (2020) [32]and Moreno de Luca (2020) [30] demonstrated diverse rates of completion for genetic testing among individuals with ASD, with 59.8% and 16.5% of subjects, respectively, undergoing testing. Conversely, Codina-Sola (2017) [34] in Spain found low rates of utilization, with only 30% of families visiting a genetic service and 13% of patients undergoing recommended tests. Similarly, research by Amiet (2014) [35] highlighted disparities between countries, noting significantly fewer US participants (27.8%) undergoing diagnostic genetic testing compared to French participants (61.7%). Intriguingly, both populations expressed keen interest in genetic screening for autism. In Sweden, Hellquist and Tamminies [33] reported low referral rates for clinical genetic testing post-ASD diagnosis, particularly among autistic children (9.1%) and adolescents/adults (2.8%), with higher rates observed in those with intellectual disability and language disorders. These findings emphasize the imperative for enhanced access to and utilization of genetic testing services worldwide, aiming to facilitate early diagnosis, tailored treatment, and comprehensive support for individuals with ASD and their families. Various studies have explored reasons for underutilization: recent research suggests that 50% of requests for genetic testing from patients with autism were refused, as the test was deemed non-essential for medical management [31]. Additionally, other studies have highlighted that genetic testing was considered irrelevant by families due to unclear medical recommendations [29].

The present study aims to explore the factors that may influence clinicians’ decisions about the appropriateness of requesting genetic testing in their patients with ASD, and to determine the prevalence of genetic alterations in a representative sample of 440 children diagnosed with ASD.

## Methods

### Study design and setting

We conducted a retrospective chart review of 440 participants recruited between 1 January 2020 and 30 December 2021 The study included children with a diagnosis of autism spectrum disorder (ASD) who were being followed at the Mental Health Unit of the Hospital Virgen del Rocío in Seville, as part of the evaluation prior to the implementation of a protocol to improve the clinical care of children with ASD in 2020 and 2021. All patients suspected of having ASD by primary care pediatricians, early care, or neuropediatric units are referred to the Mental Health Unit of the Hospital Virgen del Rocío in Seville. All patients with a confirmed ASD diagnosis during 2020 and 2021 were included in this study. The HUVR has a coverage area of ​​over 800,000 inhabitants with high socioeconomic variability and is considered a Regional Reference Centre for neurological and psychiatric diseases in the South of Spain (Andalusia), and the Mental Health Unit centralizes the monitoring of a high volume of patients with autism from many other regions of Andalusia. We looked at the number of children with a diagnosis of Genetically Tested Autism Spectrum Disorder GTASD compared with those Non- Genetically Tested Autism Spectrum Disorder NGTASD. This programme was approved by the local institutional review board (the Clinical Research Ethics Committee of Andalusia) in accordance with international research ethics standards.

### Clinical assessment

The diagnosis of ASD was made according to the DSM-5 criteria [3]. In cases of diagnostic complexity, the ADOS-2 test, which has been internationally validated for the diagnosis of patients with ASD, was used. The ADOS-2 is a semi-structured, 45–60 min session of observation and interaction between a clinician and the child, used to support the diagnosis of ASD [36]. Data were collected via the patient’s digital health record. We collected demographic, medical and neuropsychological information for all subjects. We also recorded the presence of a first- and second-degree family history of neurodevelopmental disorders. The presence of dysmorphic features or organic co-morbidities was also recorded. According to key clinical guidelines [28] MRI was requested in patients with dysmorphic features and/or suspicious findings (seizures, intracranial manifestations of genetic disorders, abnormal neurological examination or clinical suspicion after anamnesis or physical examination). (More details in supplementary material)

### Genetic testing

All genetic testing was prescribed by consultant pediatric neurologists, with indications noted in their medical records. No genetic sequencing was requested, for example, of *PTEN* in children with macrocephaly or *MECP2* in girls with psychomotor regression. The following are descriptions of the different genetic tests performed: (i) The CMA test employs comparative genomic hybridisation technology (Agilent CGX™ v1.1 8-plex array platform with 60 K oligonucleotide probes) on a commercial same-sex diploid DNA sample to search for insertions and/or deletions (indels, CNVs) throughout the genome. The identified variants were compared to the databases DECIPHER, ClinVar, SFARI, GenoGlyphix (Perki-Elmer), DGV, Dosage Sensitivity ClinGen, ISCA, HGMD, Autism Chromosome Rearrangement Database, Gnomad and OMIM [37–39] The variants were then classified as either pathogenic, benign, or of uncertain significance [40] (ii) The Fragile X test is designed to detect *FMR1* disorders. The main cause of the syndrome is a CGG trinucleotide repeat expansion in the 5’UTR region of the *FMR1* gene. Normal and mutated categories of *FMR1* alleles were determined in accordance with ACMG guidelines [39]. The normal repeat size is from 5 to 44 repeats, the grey zone ranges from 45 to 54, premutation is from 55 to 200, and full mutation is greater than 200. (iii) Karyotype analysis is conducted to evaluate the number and structural aspects of chromosomes. Standard methods were utilised for cell culture and subsequent analysis using GTG banding and/or fluorescence in situ hybridisation (FISH) [41]. (iv) We also identified a subset of patients for whom a clinical exome was performed, which is not a routine test. Exome sequencing is conducted to obtain quantitative and qualitative extraction and evaluation of the DNA sample. This involves capturing and enriching the exonic regions and flanking intronic areas of the genes contained in the SureSelect Exome v6 sequencing panel from Agilent, which comprises of over 20,000 genes. Variants of interest located within the exonic and intronic regions, up to +/-10 nucleotides of the studied genes, were identified with respect to the reference genome (hg19). The variants were filtered based on specific quality criteria, including call quality > 20, coverage > 10x, genotype quality > 20, and allele fraction > 20. All findings were classified according ACMG recommendations [42]. Any finding was classified as variant with a frequency above 1%. All findings were interpreted based on the consultation of different databases. (See supplementary material)

### Data Analysis

Initially, we determined the percentage of genetic exams requested (karyotype, CMA array, fragile X, exome sequencing) in the overall sample and compared the findings with the primary clinical guidelines [39]. Subsequently, we separated the sample into two subsets: GTASD and NGTASD individuals were compared based on demographic and clinical assessment data, including age at diagnosis, sex, urban or rural area, family history of neurodevelopmental disorders, diagnostic complexity, dysmorphic features, and organic comorbidities. Non-clinical factors such as the time from referral to the child and adolescent mental health unit and age at enrollment were also evaluated. We aimed to identify any significant differences between the two groups. We calculated descriptive statistics for all variables and determined correlations and statistical significance using chi-squared and t-tests for independent groups. We identified and compared the language and intellectual functioning characteristics of individuals with genetic changes and those without. In order to further specify the findings of the univariate analysis, we conducted two binary logistic regression analyses. The first analysis used ‘being genetically tested’ as the outcome and considered the remaining variables as exposures simultaneously. The second analysis used ‘individual with genetic alteration’ as the outcome and considered the remaining variables as exposures simultaneously. To provide a more detailed qualitative description, we created tables of findings for individuals with genetic alterations. Statistical significance was established at a 2-sided P value of less than 0.05. The Statistical Package for Social Sciences (IBM SPSS, version 28.0, Armonk, NY: IBM Corp) was utilised for the analysis.

## Results

### Demographic and clinical factors

The age of the four hundred forty participants with a confirmed diagnosis of ASD ranged from 2 to 18 years, and the gender difference ratio is approximately 5:1. One hundred eighty-four children (41.8%) were from rural areas. The mean age at diagnosis was 37 months, 110 children (25.0%) had a family history of NDD, 132 (30.0%) of the patients had dysmorphic features and organic comorbidities, 159 (36.1%) were referred to a complex diagnostic service, and the mean referral time to specialist services was 22 months. (Table 1a)

**Table 1a:** Genetically tested ASD individuals GTASD vs non genetically tested ASD individuals NGTASD

	Univariate analysis					Multivariate analysis 95% C.I				
	All *n* = 440	GTASD *n* = 246	NGTASD *n* = 194	F	p value	OR	Lower	Upper	p value	
Gender (male)	369 (83.9)	204 (82.9)	165 (85.1)	x^2^ = 0.268	0.605	0.872	0.460	1.654	0.675	
Demographics (rural)	184 (41.8)	111 (45.1)	73 (37.6)	x^2^ = 2.611	0.129	0.702	0.447	1.102	0.124	
Family history of NDD	110 (25.0)	65 (26.4)	45 (23.2)	x^2^ = 0.505	0.356	0.777	0.463	1.302	0.338	
Dysmorphic features and/or organic comorbidities	132 (30.0)	104 (42.3)	28 (14.4)	x^2^ = 38.55	0.001*	0.166	0.095	0.288	< 0.001*	
Diagnostic complexity	159 (36.1)	88(35.6)	71 (36.6)	x^2^ = 0.015	0.981	1.485	0.913	2.414	0.111	
Age at enrollment. Mean (SD)	8.8 (4.5)	7.5 (3.7)	10.5 (4.9)	t = 26.42	0.0001*	1.103	1.025	1.188	0.009*	
Age (months) at ASD diagnosis. Mean (SD)	71 (37)	59.5(27)	85.5(41)	t = 39	0.0001*	0.984	0.970	0.998	0.025*	
Referral time to specialized services (months) Mean (SD)	10(22)	9.8 (17)	8.9 (23)	t = 0.145	0.449	1.020	1.010	1.031	< 0.001*	

In the univariate analysis p- values are for independent sample t tests for continuous dependent variables or χ2 tests whenever both variables were categorical

*NDD* Neurodevelopmental disorders, *ASD* Autism Spectrum disorder, *GTASD* Genetically Tested Autism Spectrum Disorder, *NGTASD* Non-genetically tested Autism Spectrum Disorder.  *p < 0.05

### Genetically tested ASD

Of the total sample, 246 patients (56%) were genetically tested with at least one genetic study. The most commonly requested genetic test was CMA, which was performed in 186 patients (42% of the total sample) with normal results in 167 patients. Of these 167 patients, 37 (20%) underwent a clinical exome and in 17 of these patients an alteration was found. Only 100 of 369 (28%) of men received both recommended tests (Fragile X and CMA). Unfortunately, we did not find any objective data explicitly justifying when to request of a genetic study and the request of one versus several tests. Regarding the factors related to the decision about the appropriateness of genetic testing, we found statistical differences in the proportion of dysmorphic features and organic comorbidities between GTASD and NGTASD *(42.3% vs. 14.4%, F:38.55, p = 0. 001);* we also found that the mean age of ASD diagnosis was lower in the GTASD group than in the NGTASD group *(59.5, SD 27 months vs. 85.5, SD 41, t student 39 p = 0.001)* and that the mean age of enrollment in specialist services was lower in the GTASD group than in the NGTASD group *(7.5, SD 3.7 years vs. 10.5, SD 4.9, t student 26.42 p = 0.001)*. There were no differences in demographic factors, family history of NDDs, diagnostic complexity or referral time to specialist services between the GTASD and NGTASD groups. Similarly, in the multivariate analysis, a binary logistic regression model was employed to assess the combined effects of various factors on genetic testing for ASD. In this comprehensive analysis, dysmorphic features and/or organic comorbidities remained significantly associated with genetic testing for ASD *(OR:0.166, 95%C.I.:0.095–0.288*, *p* *< 0.001).* Additionally, age at enrollment *(OR:1.103, 95% C.I.:1.025–1.188, p* *= 0.009)*, age at ASD diagnosis *(OR:0.984, 95% C.I.:0.970–0.998, p* = *0.025)*, and referral time to specialized services *(OR:1.020, 95% C.I*: *1.010–1.031, p* *< 0.001)* emerged as significant predictors of genetic testing for ASD. These findings underscore the influence of specific clinical characteristics, particularly the presence of dysmorphic features and/or organic comorbidities, on the decision-making process regarding genetic testing among individuals with ASD.

### Clinical characteristics in individuals with genetic alterations vs individuals without genetic alterations

Children with genetic alterations (*n* = 43) were more frequently men than women *(72.1% n = 31 vs. 27.9% n = 12.* Moreover, children with genetic alterations presented more frequently intellectual disability *(62.8% n = 27 vs. 33.5% n = 68; F 13.472; p = 0.001*) and dysmorphic features *(60.5% n = 26 vs. 38.5% n = 78; F 7.064; p = 0.008*) than those without genetic alterations (*n* = 203). In the multivariate analysis, we examined the combined effects of various clinical factors on the likelihood of having genetic alterations among individuals with ASD. The analysis revealed several significant associations. Specifically, individuals with intellectual disability were significantly more likely to have genetic alterations compared to those without intellectual disability *(OR:3.654, 95% C.I.:1.573–8.491, p* = *0.003)*. Similarly, the presence of dysmorphic features and/or organic comorbidities was significantly associated with an increased likelihood of genetic alterations* (OR:2.120, 95% C.I.:1.945–4.299*, *p* = *0.037)*. However, no significant associations were found between genetic alterations and verbal disability, diagnostic complexity, or sex in the multivariate model. These findings suggest that specific clinical characteristics, such as intellectual disability and dysmorphic features, may serve as potential indicators for identifying individuals with ASD who are more likely to have genetic alterations (Table 1b).

**Table 1b:** Clinical factors associated with genetic alterations

	Univariate analysis					Multivariate analysis 95% C.I				
	All *n* = 246	Individuals with genetic alterations *n* = 43	Individuals without genetic alterations *n* = 194	F	p value	OR	Lower	Upper	p value	
Intellectual disability	95 (38.6)	27 (62.8)	68 (33.5)	13.472	0.001*	3.654	1.573	8.491	0.003*	
Verbal Disability	116 (47.2)	24 (44.8)	92 (45.3)	1.568	0.210	0.711	0.305	1.658	0.429	
Dysmorphic features and/or organic comorbidities	104 (42.3)	26 (60.5)	78 (38.5)	7.064	0.008*	0.472	0.233	0.957	0.037*	
Diagnostic complexity	88 (35.8)	17 (39.5)	71(35)	0.321	0.571	0.777	0.378	1.599	0.493	
Male	204 (82.9)	31(72.1)	173 (85.2)	4.320	0.036*	1.705	0.752	3.867	0.202	

In the univariate analysis p- values are for independent sample t tests for continuous dependent variables or χ2 tests whenever both variables were categorical. *p < 0.05

### Genetic alterations

From the 246 GTASD individuals 17.5% (*n* = 43) had genetic alterations, variants of unknown significance or pathogenic findings. Of those 43 patients, 46% ( *n* = 20) had alterations on CMA, 39% (*n* = 17) had alterations on exome, 9.3% (*n* = 4) had chromosome abnormalities and 9.3% (*n* = 4) had pathogenic fragile X findings. There were two patients with both exome sequencing and altered CMA.

Medical. recommendations in response to the genetic finding such referral to medical subspecialties (e.g. nephrology because of associated renal disorders in a case of kidney dysplasia) and screening for associated comorbidities were made for 43% of patients with pathogenic genetic findings.

### Genetic testing yield

The genetic testing yield including CMA, fragile X (males), karyotype, and exome was 17.47% including pathogenic mutations and mutations of uncertain significance and 12.2% including only pathogenic ones, a result similar to other studies [26]. However, when we calculated the genetic testing yield excluding exome, the yield decreased to 7.3%. It is important to clarify that exome sequencing was only performed on a very selected sample of patients who presented normal CMA but additional dysmorphic features or comorbidities besides autism. The specific CMA yield including only pathogenic mutations was 5.4% (10/186). Another 10 mutations were classified as uncertain significance (VUS) after their study by geneticists in multiple databases [43–45](Table 2). It should be noted that some of the variants of uncertain significance may be determined to be pathogenic in the future [28, 46]. The most common CNVs in this sample were deletions or duplications on 15q(*n* = 5), 16p (*n* = 2) and 17q (*n* = 2). With respect to the karyotype, the yield was 4% (4/127) (Table 3). Only 1 alteration, 46,XX,del(10)(q26.13-q26.3) was reported as uncertain significance. Three inversions were observed, a normal variant inv(2) and two apparently balanced chromosome inversion. The fragile X yield in the male population was 2% (3/142 performed). Among the 4 subjects with FMR1 diseases findings, there were 3 full mutations (FRAXA), and 1 premutation (fragile X-associated tremor/ataxia syndrome (FXTAS) (Table 4). The specific exome yield was 32.4% (12/37). Twenty seven mutations were found in 17 different patients, from those 27 only 5 were reported as pathogenic mutations and 7 were reported as probably pathogenic mutations. No mutated gene appeared in two different subjects (Table 5).

**Table 2:** Results of chromosomal microarray analysis CMA

Copy number variant	Coordinates	Size	Inheritance	Signification
16p13.11	Duplication	1.16-1.81 Mb	NA	Pathogenic
15q11.2 ^1^	Duplication	263.2 Kb -23.72 Mb	NA	Uncertain
17q12 × 1 ^1^	Duplication	1.77–2.12 Mb	NA	Pathogenic
3p26.3-p26.2	Duplication	345.24- 380.77 Kb	NA	Pathogenic
16p12.2	Duplication	167.83- 426.77 Kb	Maternal	Uncertain
2p24.3-p24.1	Duplication	5.71 Mb	Novo	Pathogenic
2q13	Duplication	1.66–2.23 Mb	Novo	Pathogenic
15q11.2-q13.1	Duplication	4.80–6.13 Mb	NA	Pathogenic
15q13.3	Duplication	409,39 Kb-1,02 Mb	NA	Uncertain
15q13.3	Duplication	409,39 Kb-1,02 Mb	NA	Uncertain
4p16.3	Deletion	448.28 Kb-541.29 kb	NA	Uncertain
8q24 ^2^	Duplication	NA	NA	Uncertain
11q11 ^2^	Deletion	NA	NA	Uncertain
Xq26.1-q26.2	Duplication	622.61-838.26 Kb	NA	Uncertain
17q12	Deletion	1.77–2.12 Mb	Novo	Pathogenic
4q23	Duplication	0,336 Mb	NA	Pathogenic
17p 12	Deletion	1.32–1.56 Mb	NA	Pathogenic
10q26.13-q26.3	Deletion	8.35 MB	Novo	Pathogenic
5q12.1	Duplication	1.07–1.27 Mb	NA	Uncertain
8p23.2	Deletion	447.07–625.15 kb	NA	Uncertain
15q13.3	Duplication	409.39 kb- 1.02 Mb	NA	Uncertain
13q12.11	Deletion	NA	Paternal	Uncertain

NA, Not available; Novo, not observed in blood sample from either parent; U, Unknown

^1^ The variants with the same number were found in the same individual respectively

**Table 3:** Results of Karyotype

Age at Diagnosis	Sex	Phenotype	Karyotype formulation	del/inv	Inheritance	Significance
4	M	Normal	46,XY,inv(2) (p11.2q13)	Inv	NA	Normal variant
6	F	Dysmorphic features	46,XX.ish del(10)(q26.13-q26.3)(D10S2290)	Del	*de novo*	Pathogenic
4	M	Normal	46,XY,inv(1)(p36.3q43)	Inv	Maternal	Apparently balanced chromosome inversion
3	F	Normal	46,XY,inv(4)(p14q12)	Inv	Paternal	Apparently balanced chromosome inversion

*Notes* M indicates male, *F* female, *inv* inversion of chromosome region, *del* deletion of chromosomal material, *NA* Not available, *de novo* not observed in blood sample from either parent, *CMA* chromosomal microarray analysis

**Table 4:** Results of FMR1 gen alterations

Type	Number of repetitions	Significance
Expansion	> 200	FRAXA
Pre- mutation	83	Fragile X-associated tremor/ataxia syndrome (FXTAS)
Expansion	> 200	FRAXA
Expansion	> 200	FRAXA

**Table 5:** Results of alterations in whole-exome sequencing

Gene name	Variant	Phenotype	Inheritance	Significance	Microarray
*CUX1* ^*1*^	c.1076 + 9G > A	Polydactyly kidney dysplasia	*de novo*	Uncertain	Normal
*ZNF711* ^*1*^	c.779-3 A > G		Maternal	Uncertain	Normal
*GPC4* ^*1*^	c.140 C > T p.(Ala47Val)		Maternal	Uncertain	Normal
*CIC* ^*1*^	c.5279 C > T p.(Ala1760Val)		Maternal	Uncertain	Normal
*ATRX2* ^*2*^	c.2991 C > G	Polydactyly ocular anomalies	*de novo*	Uncertain	Normal
*PMM2* ^*2*^	c.470T > C		*de novo*	Pathogenic	Normal
*CHAMP1* ^*3*^	c.325 C > T	Normal	*de novo*	Probably pathogenic	Normal
*SCN1A* ^*3*^	c.2201T > G.		*de novo*	Uncertain	Normal
*TCF12* ^*4*^	c.546dup	Paroxysmal episodes	*de novo*	Uncertain	Normal
*GNAO1* ^*4*^	c.563 C > T		*de novo*	Probably pathogenic	Normal
*CTCF* ^*5*^	c.1118 A > T	Microcephaly	*de novo*	Probably pathogenic	Normal
*FLG* ^*5*^	c.1501 C > T		*de novo*	Pathogenic	Normal
*SOX6*	c.949G > C	Normal	Maternal	Uncertain	Normal
*USP9X*	c.6077 C > T	Normal	Maternal	Uncertain	Normal
*UPF3B*	c.548 A > T	Macrocephaly	Maternal	Pathogenic	Normal
*CUX2*	c.79G > T	Epileptic encephalopathy	NA	Pathogenic	Normal
*SETD5*	c.3327 C > A	Normal	Paternal	Uncertain	Normal
*UBN2*	c.1315 C›G	Macrocephaly	NA	Uncertain	Normal
*CDKL5*	c.100-3 C > G	Normal	NA	Uncertain	Normal
*SHANK3*	c.2576 C > T	hyperchromic spots	NA	Uncertain	Normal
*PPP2R5D* ^*6*^	c.751G > C	dysmorphic features	NA	Probably pathogenic	Normal
*MED12L* ^*6*^	c.3713 A > G; p		NA	Probably pathogenic	Normal
*GRIN1* ^*7*^	c.650G > A		Maternal	Probably pathogenic	Normal
*PNKP* ^*7*^	c.1029 + 2T > C	Hyperphagia, dysmorphic features, fail thriving	NA	Uncertain	Normal
*SETBP1* ^*7*^	c.901 C > T		NA	Uncertain	Normal
*CIC*	c.6746 A > C	Normal	NA	Probably pathogenic	Normal
*PTEN*	c.802-1G > A	Macrocephaly	NA	Pathogenic	Normal

*NA* Not available, *de novo* not observed in blood sample from either parent

^1,2,3,4,5,6^ The variants with the same number were found in the same individual respectively

## Discussion

After analyzing the factors related to clinicians’ decisions regarding genetic testing requests in a representative sample of children with ASD, we found that: (i) even though the primary guidelines [25, 28] recommend testing for all children diagnosed with ASD, only 56% of them were genetically tested. Furthermore, the prevalence of genetic alterations was 17.5% among those who were tested, including pathogenic variants and variants of uncertain significance. These changes were more commonly linked to intellectual disability and physical abnormalities. (ii) We couldn’t find explicit objective evidence to support or oppose genetic testing, nor to justify requesting one genetic assessment over multiple. (iii) Notably, only 28% of males were tested using the suggested tests (Fragile X and CMA). (iv) Children exhibiting physical abnormalities and organic comorbidities were more likely to be genetically studied than those without. (v) Prior diagnosis of ASD and enrollment in specialist services were also linked to ASD genetic testing. (vi) The presence of dysmorphic features and/or organic comorbidities, as well as intellectual disability was significantly associated with an increased likelihood of genetic alterations.

At the moment, there is no clinical or demographic data available to help us identify patients or phenotypes suitable for genetic test, and the request is driven solely by medical decision. However, referring to other international studies [47], it is advisable to recommend genetic testing for all children diagnosed with ASD. In fact, exome sequencing has been suggested as a primary test before or at the same time as CMA, due to its higher molecular diagnostic yield for neurodevelopmental disorders compared to CMA [20, 21], although in our study it was only performed in selected patients and after a normal CMA. The testing completion rate of 56% in our sample aligns with data reported in the USA and France [32, 35]. Notably, it exceeds the rate reported in a Swedish study [33]. However, it’s important to note that the Swedish study’s reliance on community survey responses may introduce biases related to respondent characteristics, accuracy, understanding, and non-response.

Our findings surpass the completion rate reported in Spain seven years ago [34], this could be attributed to the improved utilization of genetic testing in our hospital, which is recognized as a Regional Reference Centre for neurological and psychiatric diseases in the South of Spain (Andalusia). Additionally, it may reflect the presence of more informed families and patient associations actively advocating for comprehensive healthcare services. In Spain, the national guideline supports the request of a genetic testing for ASD patients, which typically includes CMA, FRAXA, and Karyotype tests in this specific order. However, the application of exomes or other NGS techniques varies depending on the region and their accessibility. Due to the limited availability of certain tests across different regions of Spain, there exists a significant disparity in their utilization, resulting in an evident underutilization of genetic services, as reported previously [34]. Moreover, in many cases, there is no standardized approach, and not all ASD patients undergo the same genetic tests across all centres. To address this disparity, a strategic plan named IMPacT [48] has been developed recently to advance precision medicine in Spain, with the goal of enhancing equity among regions and patients within the Spanish healthcare system. This initiative could also account for the observed rise in the utilization of genetic tests in our study compared to previous Spanish research. Ongoing efforts in this direction are crucial to achieve full equity nationwide and to ensure the implementation of these protocols across all regions.

Among these GTASD, the diagnostic yield of 7.3%, apart from exome, is slightly lower with previous studies which have reported an overall diagnostic yield of around 10–20% [25, 46, 49]. Our findings align with previous WHO data [49, 50], as there were as disparities were observed when examining clinical features such as intellectual disability and dysmorphic features and/or organic comorbidities between children with and without pathogenic findings in genetic testing. Likewise, we found that these patients are more likely to undergo genetic testing than those without dysmorphic features. Similarly, patients with complex diagnoses and those referred earlier to specialist autism services are more likely to receive genetic testing than those with more obvious clinical criteria for ASD diagnosed in primary care.

In terms of clinical factors, our findings are consistent with the scientific literature as we found that intellectual disability and dysmorphic features were associated with the presence of genetic alterations, but it should be noted that, as we also found, these patients are more likely to be genetically tested than patients without dysmorphic features or intellectual disability. Among the identified mutations related to intellectual disability, we found, for example, the *CIC* c.6746 A > C; p.(Lys2249Thr) an autosomal dominant mutation in the *CIC* gene associated with intellectual disability type 45 [51] and the recurrent deletion/duplication of the 15q13.3 region associated with phenotypes with different degrees of intellectual disability [52].

The specific CMA yield including only pathogenic mutations was 5.4% including only pathogenic variants, which is lower than previous studies reporting 10% [20, 49]. However, the wide variation in the utility of the test depending on the study population is evident, for example, current studies report higher performance until 25% [47] but other publications reveal definitively pathogenic CNVs using CMA in 5.4–14%, median 9% [28] and 10% using VUS and pathogenic variants [46] like our study. This may be because the detection rate and pathogenic yield of CMA varies significantly depending on the primary indications for testing, the age of the individuals tested, and the specialty of the ordering physician. A recent Spanish study [26] highlights age as a performance-modifying factor, finding a causal genetic alteration in 22.5% of patients over 5 years old, but only in 3.9% of patients under this age. This could be one of the causes of our lower yield. More studies in the Spanish population on the diagnostic yield genetic tests would be necessary to assess the specific figures in our population.

There could be many reasons that could explain why genetic studies are not requested for all ASD patients, such as the complexity of a patient’s phenotypic profile or insufficient consultation time to obtain a complete diagnosis. Recent studies [30] have found that dissonance between professional recommendations and clinical practice may be explained by limitations in clinicians’ knowledge and comfort with genetic testing, a lower frequency of genetic testing in patients diagnosed with ASD by psychiatrists and psychologists than by pediatricians, changes in genetic testing practices over time, and a reduced likelihood of testing being offered to adolescents with ASD. The ACMG and the American Academy of Pediatrics recommends offer a genetic consultation to all persons/families with ASDs, and discuss a genetic testing to all patients and families with ASDs, but not all professionals have this knowledge.

Despite the advantages and benefits of genetic testing, it is also important to take into account the limitations and potential risks of using genetic testing, as well as legal and ethical issues. Numerous publications increasingly cover these points, and emphasize the potential undesirable effects: an earlier diagnosis of ASD may not be better [53], genetic tests are aimed at curing or eliminating ASD and not at improving quality of life [54, 55], genetic research directed at the germline is not ethically correct [55], among others. Another point to highlight and that must be taken into account before performing genetic tests is the incidental finding of pathogenic genetic alterations associated with other organic or psychiatric disorders in asymptomatic people, called incidental findings [56]. It is also crucial to emphasize to families that undergoing a genetic test may reveal familial information that they may not be willing to know, such as a family predisposition to cancer or other neurological diseases that could manifest in the future. All these issues should be discussed with the family in advance to ensure that their right to know or not know is respected and not violated. Legally, there are also controversies because on many occasions genetic information is used and shared in other research without proper informed consent and can generate negative discrimination [55]. Therefore, it is important to carry out equitable, moral and ethical use of genetics so that the benefits outweigh the undesirable effects [54].

On the other hand, there are subjective or observer-dependent assessments of the changes found in CMA. Duplications in the 15q13.3 region have been associated with intellectual disability, global developmental delay, speech and language delay and, to a lesser extent, autism spectrum disorder and epilepsy. The 15q13.3 duplication presents greater clinical variability and lower penetrance than the corresponding deletion of the region. It has not been possible to associate a definitive phenotype to this duplication. Deletions at 15q11.2 BP1-BP2 have been shown to confer a slightly increased risk of ASD with low penetrance (10.4%) [57, 58]. We observed discrepancies in the way laboratories reported some results, for example, we found 5 patients with 15q duplications. The 15q11.2 BP1-BP2 duplications were reported by the genetics department on one occasion as uncertain significance and on another occasion as pathogenic, and to date there is strong evidence that the 15q11.2 BP1-BP2 duplication is associated with a modestly increased risk of ASD and with a specific pattern of non-syndromic ASD [59]. This discrepancy in interpretation of a finding between different laboratories is also found between different databases and even within the same database over time. In addition, 9 CNVs of uncertain significance have been reported. Although the impact of individual CNVs remains uncertain, their contribution to ASD risk cannot be excluded due to ongoing reclassifications and new lines of research. There are several studies showing the conversion of uncertain CNVs into pathological ones [46].

One of the key strengths of this study is the large sample. The study’s results have practical implications for clinical practitioners, suggesting that implementing protocols and algorithms could be a valuable strategy for improving the detection of genetic alterations in children with ASD. Some limitations of this study should be noted, including the absence of standardized vocabulary, such as the Human Phenotype Ontology (HPO) [60], to detail phenotypic profiles. Additionally, the information collected on the rationale for genetic study requests is based on clinical subjectivity. The lack of parental data for many of the CNVs and variants identified precludes clear categorization as pathogenic or uncertain. In our study, there are several mutations with unknown heritability due to the lack of familial segregation, which could be limitations in classifying these mutations as benign or pathogenic [61]. The geographical scope of our sample, which is primarily concentrated within a specific region of Spain, may limit the generalizability of our results beyond this particular area. While our findings provide valuable insights within this context, caution should be exercised when extrapolating these conclusions to broader populations or different geographical regions.

It is not only the phenotypic heterogeneity but also the genetic heterogeneity [62] and the low penetrance of same findings that adds to the complexity of autism and there is still a long way to go in genomic sequencing research to improve understanding of the impact of individual variants on the pathogenesis and severity of autism. While the primary cause of ASD is attributed to genetics, future research should also explore the potential role of epigenetic mechanisms, as they involve regulatory molecules like miRNAs, which may impact the risk of autism through genetic regulation [63, 64]. It is necessary to combine clinical, genomic and molecular criteria to create phenotypes of autistic patients. This would allow progress towards the creation of specific genomic models associated with each phenotype. As of now, the yield of these tests in the autistic population without dysmorphic features or intellectual disability remains uncertain. In conclusion, our findings emphasize the importance of establishing algorithms to facilitate targeted genetic consultation for individuals with ASD who are likely to benefit, considering clinical phenotypes, efficiency, ethics, and benefits.

## Electronic supplementary material

Below is the link to the electronic supplementary material.

Supplementary Material 1

## Data Availability

No datasets were generated or analysed during the current study.

## Abbreviations

NDD: Neurodevelopmental disorders
CNV: Copy Number Variants
FMR1: Fragile X Messenger Ribonucleoprotein 1
ASD: Autism Spectrum disorder
NGTASD: Non-genetically tested Autism Spectrum Disorder
GTASD: Genetically Tested Austism Spectrum Disorder
MRI: Magnetic Resonance Imaging
PTEN: Phosphatase and Tensin Homolog Gene
MCEP: Methyl- CpG-binding protein 2
CMA: Chromosomal microarray analysis
