# Supplementary material for: Exploring genetic testing requests, genetic alterations and clinical associations in a cohort of children with autism spectrum disorder
Source: Eur Child Adolesc Psychiatry. 2024 Apr 8;33(11):3829–40. doi: 10.1007/s00787-024-02413-x (PMC11588872; doi:10.1007/s00787-024-02413-x)
Supplement: Supplementary file 1 — Supplementary Material 1 [file 787_2024_2413_MOESM1_ESM.docx]

Supplementary material

| Table S1 Variables Abstracted From Medical Records | | |
| --- | --- | --- |
| Demographic Characteristics | Medical an Neuropsychological  Characteristics | Genetic, clinical and imaging tests |
| Age at diagnosis | Dysmorphic features reported by clinician | ADOS-2, ID, language alteration |
| Sex |  | *Karyotype, X fragile, Microarray, exome* |
| Demographics (rural/urban) | Epilepsy | MRI |
| ADOS-2: Autism Diagnostic Observation Schedule, Second Edition ; MRI: Magnetic Resonance Imaging; ID: Intellectual disability | | |

| Table S2 MRI anomalies reported in patients with dysmorphic features and/or organic comorbidities | | |
| --- | --- | --- |
| Nonspecific anomalies | 19 | |
|  | Hyperintense white matter signal foci: delayed myelination | 9 |
|  | Choroid plexus cysts | 2 |
|  | Left hippocampal malrotation | 2 |
|  | Megacysterna magna | 4 |
| Significant alterations |  | |
|  | Old ventricular hemorrhage, leukoencephalomalacia | 2 |

| Table S3. Genetic Databases used | |
| --- | --- |
| Database Name | Description |
| DECIPHER | Database of Chromosomal Imbalance and Phenotype in Humans Using Ensembled Resources |
| ClinVar | Archive of reports on relationships among human variations and phenotypes, with a focus on clinical significance |
| SFARI | Simons Foundation Autism Research Initiative - Resource for autism research community |
| GenoGlyphix (PerkinElmer) | Tool for visualizing and interpreting genomic data provided by PerkinElmer |
| DGV (Database of Genomic Variants) | Catalog of structural variations in the human genome |
| Dosage Sensitivity ClinGen | Part of ClinGen project, focuses on curating and interpreting dosage sensitivity information for genes |
| ISCA | International Standards for Cytogenomic Arrays - Database for curated and standardized genomic variant information |
| HGMD | Human Gene Mutation Database - Collection of information on human gene mutations associated with inherited diseases |
| Autism Chromosome Rearrangement Database | Database dedicated to cataloging and curating chromosomal rearrangements associated with autism spectrum disorders |
| gnomAD | Genome Aggregation Database - Large-scale genome and exome database aggregating genetic variation data |
